# Is the “golden age” of the “golden hour” in sepsis over?

**DOI:** 10.1186/s13054-015-1167-3

**Published:** 2015-12-29

**Authors:** Derek S. Wheeler

**Affiliations:** Division of Critical Care Medicine, Cincinnati Children’s Hospital Medical Center, 3333 Burnet Avenue, Cincinnati, OH 45229-3039 USA; Department of Pediatrics, University of Cincinnati College of Medicine, Cincinnati, OH USA

## Abstract

The so-called “golden hour” of trauma resuscitation has been applied to a number of disease conditions in the intensive care unit (ICU) setting. For example, the “golden hour” as applied to the treatment of critically children and adults with severe sepsis and septic shock is based upon early recognition, early administration of antibiotics, and early reversal of the shock state. However, several clinical studies published over the last decade have called into question this time-honored approach and suggest that overly aggressive fluid resuscitation may cause more harm than good. Perhaps we are finally leaving the “Golden Age” of the “golden hour” and entering a new age in which we are able to use a more personalized approach to fluid management for patients with severe sepsis/septic shock.

For nearly two decades, if not longer, the treatment of critically ill children with severe sepsis and septic shock has rested on three fundamental tenets—early recognition, early administration of antibiotics, and early reversal of the shock state with aggressive fluid resuscitation and administration of vasoactive medications. Recently, these fundamental tenets of sepsis treatment have been called into question. Investigators from the Alberta Sepsis Network prospectively enrolled 79 critically ill children with severe sepsis/septic shock admitted to two pediatric intensive care units (PICUs) over a 2-year period. Aggressive fluid resuscitation in the early stages of treatment was independently associated with longer PICU length of stay and days on mechanical ventilation [1]. While the Alberta Sepsis Network investigators were careful to not to overstate their conclusions, the results of this study further call into question what was once considered part and parcel of the management of critically ill children with severe sepsis and septic shock.

A vast body of literature strongly supports the paradigm that early initiation of therapy in the intensive care unit (ICU) setting, regardless of the clinical condition, is associated with significantly improved outcomes. Indeed, the so-called “golden hour” for resuscitation of trauma patients has been applied to a number of clinical conditions, including acute coronary syndrome, stroke, and severe sepsis/septic shock. Early recognition and treatment of these and similar conditions is believed to arrest the complex chain of events occurring at the molecular and cellular levels that lead to irreversible organ dysfunction and eventual death. The concept of a “golden hour” in patients with severe sepsis/septic shock was supported by early studies performed in both critically ill children [2–4] and adults [5]. The landmark early, goal-directed therapy in sepsis trial [5], in particular, supported the concept of a “golden hour” as one of the first prospective, randomized, clinical trials in sepsis to show a significant reduction in overall mortality. With the subsequent work of the Surviving Sepsis Campaign [6], it seemed that the field of critical care medicine had truly entered a “golden age” of the “golden hour” of sepsis treatment.

Several small, retrospective and prospective, observational studies performed in critically ill children have demonstrated an association between fluid overload and poor outcomes. While the initial studies involved critically ill children requiring renal replacement therapy, subsequent studies showed that fluid overload was associated with worse outcomes even in critically ill children who were not receiving renal replacement therapy [7, 8]. The Fluid and Catheters Treatment Trial (FACTT), a prospective, randomized, clinical trial using a 2 × 2 factorial design, showed that a restrictive fluid management strategy was superior to a liberal fluid management strategy in 1000 critically ill adults with acute lung injury [9]. The mean cumulative fluid balance during the first 7 days of treatment was −136 mL in the restrictive fluid management group versus 6992 mL in the liberal fluid management group. While 60-day mortality was not different between the two groups, the number of ventilator-free days and ICU-free days was higher in the restrictive fluid management group, with no significant differences in the incidence or prevalence of shock or use of dialysis in the first 60 days. Over 3000 African children (Uganda, Kenya, and Tanzania) presenting with severe febrile illness and clinical evidence of impaired perfusion were randomized to receive boluses of 0.9 % saline (20–40 mL/kg), 5 % albumin (20–40 mL/kg), or no bolus at initial presentation. Somewhat surprisingly, children randomized to the control group (no bolus) had lower mortality at 48 hours and 4 weeks after treatment. Notably, patients with severe hypotension were randomized to either the saline or albumin group only [10]. These significant differences in mortality between the two bolus groups versus no bolus control group occurred in children regardless of initial presentation among three pre-defined clinical presentations (shock, respiratory, or neurologic disease), and the cause of death in the bolus groups was more often attributed to cardiovascular collapse rather than fluid overload [11]. Finally, in the last 2 years, several prospective, randomized, multicenter clinical trials have failed to demonstrate any improvement in outcomes with protocolized, early goal-directed therapy of critically ill patients with severe sepsis/septic shock [12].

With all of the aforementioned studies in mind, the results provided by the Alberta Sepsis Network investigators provide additional evidence that overly aggressive fluid resuscitation in critically ill children with severe sepsis/septic shock is not only unlikely to be helpful, but also is probably harmful. We simply do not have the ability to adequately discriminate between patients who are likely to benefit from fluid resuscitation versus those who are likely to be harmed from fluid resuscitation, given that the relevant pathophysiology of shock occurs at the molecular, cellular, and organ-specific levels. To this end, a panel of biomarkers has been used to stratify the risk of mortality for critically ill children with severe sepsis/septic shock, and a recently published study showed that a positive fluid balance after ICU admission is associated with worse outcomes for those children with a low-risk of mortality, but not in those children with moderate-to-high risk of mortality [13].

Rather than abandoning a time-honored therapeutic strategy, perhaps we should first strive to find better clinical markers of fluid-responsiveness. Clearly, the traditional markers used to date (heart rate, urine output, blood pressure, capillary refill, central venous pressure, pulmonary artery occlusion pressure, etc.) are not sufficient for the task. We need better therapeutic endpoints of resuscitation that are readily available at the bedside. Until that time, it seems prudent to recommend a careful, deliberate approach to fluid resuscitation [14]. Critically ill children with low blood pressure should be appropriately resuscitated with fluid bolus therapy (20–40 mL/kg, repeated as necessary until blood pressure normalizes). However, beyond the initial phase of resuscitation (and in the absence of severe hypotension), fluids should be carefully titrated in small, incremental doses (“fluid challenge”), perhaps as small as 3–5 mL/kg in children, based upon the currently available therapeutic endpoints [14, 15]. Additional fluids should be withheld in the absence of clinically significant improvement in these endpoints. Perhaps we are leaving the “golden age” of the “golden hour” and entering a new age in which we are able to use a more personalized approach to the treatment of severe sepsis/septic shock.

## Abbreviations

ICU: Intensive care unit
PICU: Pediatric intensive care unit
